# The Antimicrobials Anacardic Acid and Curcumin Are Not-Competitive Inhibitors of Gram-Positive Bacterial Pathogenic Glyceraldehyde-3-Phosphate Dehydrogenase by a Mechanism Unrelated to Human C5a Anaphylatoxin Binding

**DOI:** 10.3389/fmicb.2019.00326

**Published:** 2019-02-26

**Authors:** Sara Gómez, Javier Querol-García, Gara Sánchez-Barrón, Marta Subias, Àlex González-Alsina, Virginia Franco-Hidalgo, Sebastián Albertí, Santiago Rodríguez de Córdoba, Francisco J. Fernández, M. Cristina Vega

**Affiliations:** ^1^Center for Biological Research, Spanish National Research Council, Madrid, Spain; ^2^CIBER de Enfermedades Raras, Madrid, Spain; ^3^Institut Universitari d’Investigació en Ciències de la Salut, University of the Balearic Islands, Mallorca, Spain

**Keywords:** GAPDH – glyceraldehyde-3-phosphate dehydrogenase, *Streptococcus pyogenes*, *Clostridium perfringens*, anacardic acid, curcumin, complement – immunological term, enzyme inhibition, X-ray crystallography

## Abstract

The ubiquitous and highly abundant glycolytic enzyme D-glyceraldehyde-3-phosphate dehydrogenase (GAPDH) is pivotal for the energy and carbon metabolism of most organisms, including human pathogenic bacteria. For bacteria that depend mostly on glycolysis for survival, GAPDH is an attractive target for inhibitor discovery. The availability of high-resolution structures of GAPDH from various pathogenic bacteria is central to the discovery of new antibacterial compounds. We have determined the X-ray crystal structures of two new GAPDH enzymes from Gram-positive bacterial pathogens, *Streptococcus pyogenes* and *Clostridium perfringens*. These two structures, and the recent structure of *Atopobium vaginae* GAPDH, reveal details in the active site that can be exploited for the design of novel inhibitors based on naturally occurring molecules. Two such molecules, anacardic acid and curcumin, have been found to counter bacterial infection in clinical settings, although the cellular targets responsible for their antimicrobial properties remain unknown. We show that both anacardic acid and curcumin inhibit GAPDH from two bacterial pathogens through uncompetitive and non-competitive mechanisms, suggesting GAPDH as a relevant pharmaceutical target for antibacterial development. Inhibition of GAPDH by anacardic acid and curcumin seems to be unrelated to the immune evasion function of pathogenic bacterial GAPDH, since neither natural compound interfere with binding to the human C5a anaphylatoxin.

## Introduction

D-Glyceraldehyde-3-phosphate dehydrogenase (GAPDH, E.C. 1.2.1.12) catalyzes the sixth glycolytic reaction (Supplementary Figure S1). Before GAPDH, the previous reactions of glycolysis consume energy in the form of two ATP molecules; in contrast, the following reactions generate four ATP molecules so that glycolysis has a net energy-producing output. Therefore, GAPDH appears as one of the most promising enzymes to inhibit in order to interfere with glycolysis, since blocking the GAPDH-catalyzed reaction would still allow the cells to consume energy without being able to regenerate it. For organisms that rely on glycolysis for all or most of their energy generating reactions, inhibiting GAPDH offers a unique opportunity. For example, trypanosomatids thrive exclusively on glycolysis as it is performed in a specialized organelle and a multitude of inhibitors have been developed that are efficient at combating leishmaniosis (Suresh et al., 2001), Chagas’ disease (Souza et al., 1998) and other trypanosomiases (Lambeir et al., 1991; Vellieux et al., 1993; Verlinde et al., 1994; Kim et al., 1995; Bressi et al., 2001). These inhibitors have been designed to be highly selective for the pathogen’s GAPDH in order to avoid being trapped by the abundant liver GAPDH.

The lessons learned in these inhibitor development campaigns can be transferred to the search for better inhibitors toward GAPDH of bacterial pathogens, some of which are greatly reliant on the glycolysis. GAPDH shares an evolutionarily conserved fold consisting in two juxtaposed domains, an N-terminal NAD-binding domain (NBD) and a C-terminal catalytic domain. At the boundary between the two domains lies a catalytic cysteine residue. The cleft formed by the domain interface helps to stabilize the cofactor binding pocket and provides interactions with the substrates and products. GAPDH requires the nicotinamide adenine dinucleotide (NAD+) cofactor that remains tightly bound in the active site. The active site of GAPDH contains also two structurally distinct phosphate-binding sites, the P_i_ and P_s_ sites, where inorganic phosphate and the phosphate group from the glyceraldehyde-3-phosphate (G3P) substrate bind, respectively, during reaction (Skarzyński and Wonacott, 1988; Yun et al., 2000). In fact, there are two different P_i_ sites depending on the precise location within the active site of the inorganic phosphate moiety, which are termed the “classical” and “new” P_i_ sites (Yun et al., 2000; Mukherjee et al., 2010).

Besides its glycolytic role, GAPDH assumes many diverse moonlighting functions both in eukaryotes and prokaryotes. In these non-glycolytic functions, GAPDH participates in critical cellular processes like apoptosis and replication and it has been involved in the development of human diseases and tumorigenesis. In pathogenic bacteria, GAPDH takes on virulence and immunoevasive roles that require GAPDH to be exported to the extracellular space, either associated with cell wall structures or completely detached from the GAPDH exporting bacterial cells (Sirover, 1997, 2005, 2011, 2012, 2014; Chauhan et al., 2017; Raj et al., 2017). The interaction of pathogenic bacterial GAPDH with complement components has recently emerged as an immune evasive mechanism that may be relevant for infection biology and for the therapeutic management of infectious diseases (Terao et al., 2006; Querol-García et al., 2017). Inhibition of the glycolytic or the moonlighting functions of GAPDH has been posited as a valuable strategy to curb trypanosomatid and bacterial infections (e.g., Querol-García et al., 2017). Both synthetic ADP analogs and natural products have been established as antimicrobial agents targeting GAPDH (Pereira et al., 2008; Freitas et al., 2009), which has paved the way for their development as pharmaceutical lead compounds in the quest for new therapies against infectious diseases.

The recent structural determination of *Atopobium vaginae* GAPDH at 2.1-Å resolution has provided detailed information about the configuration of the active site that can be used for the discovery of novel inhibitors. In this work, we have solved two new structures of GAPDH from pathogenic Gram-positive bacteria, *Streptococcus pyogenes* and *Clostridium perfringens*, to 1.55 and 2.55 Å resolution, respectively. The structural analysis of these three GAPDH structures and their comparison with trypanosomatid and human liver GAPDH provides insight into the structural and chemical features that selective inhibitors should have.

In particular, we have investigated the inhibitory properties of two natural compounds, anacardic acid and curcumin, which are known to have antimicrobial properties against several Gram-positive pathogenic bacteria but lack a validated target (Cheng et al., 2001; Mamidyala et al., 2013; Sharma et al., 2013; Hamad and Mubofu, 2015; Hollands et al., 2016). Curcumin, in particular, has also shown promise for the treatment of precancerous cervical lesions and advanced breast cancer, uses for which it is being tested in ongoing clinical trials (Cheng et al., 2001; Ko and Moon, 2015; Karibe et al., 2018; Norouzi et al., 2018). The characterization of the inhibitory modality of anacardic acid and curcumin has revealed that they act through uncompetitive and non-competitive mechanisms. The combination of enzymatic, structural and computational chemistry approaches sheds light on the mechanism of inhibition and raises the opportunity to develop stronger, more efficient GAPDH-targeted inhibitors for antimicrobial therapy.

## Materials and Methods

### Cloning, Expression, and Purification of GAPDH

Genomic DNA from a *Clostridium perfringens* type strain (DSM-756) and a synthetic optimized cDNA for *Streptococcus pyogenes* were used as template for PCR amplification of the respective GAPDH genes, *Sp*GAPDH (UniProt Accession No. P68777) and *Cp*GAPDH (UniProt Accession No. Q0STD4/Q8XKT9). Plasmids for expression of *Cp*GAPDH and *Sp*GAPDH were constructed by restriction-ligation cloning as tobacco etch virus (TEV)-cleavable N-terminal hexahistidine tagged fusions into pETM11 (Gunter Stier, European Molecular Biology Laboratory) and pET21b (Novagen), respectively (Querol-García et al., 2017). The expression plasmids were transformed into *E. coli* Rossetta(DE3)pLysS cells (Novagen) and transformants isolated in selective LB-agar plates. Transformants from a freshly prepared plate or from a bacterial glycerol stock were used to inoculate a 40-ml overnight starter culture from which a larger culture (2 l) was initiated the next morning. The culture was allowed to grow at 37°C in LB or Power Broth (Athena) media supplemented with 100 μg/ml ampicillin (for *Cp*GAPDH) or 30 μg/ml kanamycin (for *Sp*GAPDH) and 35 μg/ml chloramphenicol to an absorbance of 0.6–0.8 at 600 nm and the temperature was lowered to 20°C for 1 h before induction. The culture was then induced with 0.5 mM isopropyl β-D-thiogalactopyranoside (IPTG) for 18 h. Cells were harvested by centrifugation at 6,000 × *g* for 20 min at 4°C and either used immediately or stored at -80°C until use. The cell pellet from 2 l of culture was resuspended in 40 ml of a buffer containing 50 mM Tris-HCl pH 8.0, 500 mM NaCl, 20 mM imidazole, and 1 mM phenylmethylsulfonyl fluoride (PMSF), and cells were lysed by sonication. The lysate was centrifuged at 20,000 × *g* for 30 min at 4°C and clarified further by filtration through a 0.22 μm membrane. The purification procedure consisted of a capture step by affinity chromatography in which the clarified lysate containing N-terminal hexahistidine enzyme was loaded on a 5-ml HisTrap column (GE Healthcare) charged with nickel chloride. GAPDH was eluted using a linear gradient of increasing imidazole concentration (250 mM) over 20 column volumes. Fractions containing GAPDH were pooled and dialyzed against a buffer containing 50 mM Tris-HCl pH 8.0, 150 mM NaCl. Finally, GAPDH was subjected to gel filtration chromatography over a HiLoad 16/60 Superdex 200 equilibrated in a buffer consisting in 10 mM Tris-HCl pH 7.5, 150 mM NaCl. We typically obtained 30–60 mg/l culture of pure *Sp*GAPDH or *Cp*GAPDH with high purity, >95% pure on a Coomassie brilliant blue-stained SDS-PAGE gel (Supplementary Figures S2, S3). Comparison of the elution volume of GAPDH with a calibration curve constructed using high and low molecular weight calibration kits (GE Healthcare), using the recommended calibration mixtures following the manufacturer’s instructions, corroborated that the quaternary structure of GAPDH corresponds to a homotetramer (Supplementary Figure S4). Finally, GAPDH was dialyzed against a buffer consisting in 10 mM Hepes-NaOH pH 7.4, 150 mM NaCl and 3.4 mM EDTA, concentrated to 30 mg/ml, dispensed in 30-μl aliquots, snap-frozen in liquid nitrogen and stored away at -80°C. *Av*GAPDH was expressed and purified using similar protocols, as previously described (Querol-García et al., 2017).

### Enzyme Kinetics

The GAPDH enzyme activity was followed spectrophotometrically by the change in absorbance at 340 nm due to NADH formation (𝜀 = 6.220 M^-1^ cm^-1^), according to a previously described method (Ferdinand, 1964). Assays were performed in an Eppendorf BioSpectrometer spectrophotometer at 25°C. A standard assay was carried out in a final volume of 0.15 ml in the presence of 40 mM Tris-HCl pH 8.5 and 2 mM EDTA, 0.1 μM *Cp*GAPDH (or *Av*GAPDH) or 0.01 μM *Sp*GAPDH and indicated concentrations of the cofactor and substrates: nicotinamide adenine dinucleotide (NAD+), G3P and inorganic phosphate (P_i_). NAD+ concentration was varied between 0.03 and 2.1 mM (while keeping fixed G3P at 5 mM and Pi at 50 mM), G3P concentration between 0.6 and 15 mM (at 2 mM NAD+ and 50 mM P_i_), and P_i_ concentration between 0.65 and 24 mM (at 2 mM NAD+ and 50 mM G3P). The reaction was initiated by addition of G3P. Michaelis–Menten parameters, *K*_M_ and *V*_max_, were obtained by non-linear regression fitting of the kinetic data using SigmaPlot v13.0 (Systat Software Inc.). Inhibition assays were performed with anacardic acid (Sigma-Aldrich A7236, PubChem ID 167551) and curcumin (Sigma-Aldrich 08511, PubChem ID 969516). Two inhibitor concentrations were tested of anacardic acid (2.5 and 5.0 μM) and curcumin (50 and 200 μM). To ascertain the inhibition modality with respect to G3P and NAD+, initial velocity measurements at each inhibitor concentration were carried out varying G3P concentration (0.6–9.6 mM) while maintaining 2 mM NAD+, or varying NAD+ concentration (0.03–2.1 mM) while maintaining 5 mM G3P (*Av*GAPDH) or 15 mM G3P (*Sp*GAPDH). In either case, potassium phosphate was kept at 50 mM.

### Enzyme-Linked Immunosorbent Assays

The interaction of *Av*GAPDH, *Sp*GAPDH, and *Cp*GAPDH with the C5a, C3a, and C3 components of the human complement system was studied with an enzyme-linked immunosorbent assay (ELISA). The wells of a polystyrene microtiter plate (F96 Maxisorp, Nunc) were coated with 100 μl of 10 μg/ml of each purified GAPDH in 50 mM Tris-HCl pH 8.5 at 4°C overnight. After coating, wells were washed with TBS-T [Tris-buffered saline pH 7.4 with 0.05% (w/v) Tween-20] and blocked with 1% (w/v) BSA (bovine serum albumin) in TBS-T for 1 h at 37°C. After washing extensively with TBS-T, various dilutions of C3 or C5a in TBS at 0.001–0.1 μM were added to the GAPDH-coated wells and the plate was incubated for 3 h at 37°C, then washed four times with TBS-T to remove unbound C3/C5a. For the binding assays carried out in presence of inhibitors, C3/C5a were supplemented with 5 μM anacardic acid or 50 μM curcumin. For detection of bound C5a/C3a/C3, a mouse anti-human (specific to C5a, C3a or C3) antibody was added to the wells at a 1:3000 dilution in TBS-T (100 μl/well) and the plate was incubated for 1 h at 37°C and washed as before. Next, a goat anti-mouse IgG-HRP conjugate (1:3000 dilution) was added, incubated for 1 h at 37°C, and washed five times with TBS-T. OPD (*o*-phenylenediamine dihydrochloride) substrate tablets (Sigma-Aldrich SigmaFast OPD tablet set, Cat. No. P9187) were dissolved in double distilled water and 200 μl added immediately per well. After 30 min incubation in the dark, the reaction was stopped with 50 μl of 3 M HCl, and the absorbance was measured at 492 nm in a microplate reader.

### Antimicrobial Susceptibility Testing

To assess the antimicrobial activity of anacardic acid and curcumin in solution, four different serotype M1 group A *Streptococcus pyogenes* strains (950383, 941079, 950358, and 950771) (Pérez-Caballero et al., 2000) were tested by broth microdilution using Todd-Hewitt broth supplemented with 0.5% (w/v) yeast extract (THY). Anacardic acid and curcumin were obtained from Sigma-Aldrich (Catalog numbers A7236 and 08511, respectively). Briefly, suspensions with a turbidity equivalent to that of a 0.5 McFarland standard were prepared by suspending the growth from overnight cultures on THY agar plates in 2 ml of sterile saline. The suspensions were further diluted 1:10 to obtain a final inoculum of 5 × 10^5^ CFU/ml. The wells of microtiter plates containing 50 μl of the bacterial suspension and 50 μl of anacardic acid or curcumin solutions at different concentrations were incubated overnight in ambient air at 37°C.

### Crystallization

Before attempting crystallization of GAPDH the required number of aliquots was quickly thawed and centrifuged at 10,000 × *g* for 10 min at 4°C to remove any potential aggregates that might have resulted from a freezing-thawing cycle. A commercial Pre-Crystallization Test (Hampton Research) was used to adjust the protein concentration to a suitable concentration for more extensive crystallization screenings, which was finally set to 7.5 mg/ml for *Sp*GAPDH and 15 mg/ml for *Cp*GAPDH. The full JCSG-plus sparse matrix and the PACT premier systematic PEG/Ion/pH screenings (Molecular Dimensions) were performed by the sitting-drop vapor-diffusion method using drops containing 1 μl either GAPDH stock supplemented with 0.6 mM NAD+ and 1 μl crystallization condition at 20°C. The optimum crystallization conditions were 0.2 M potassium nitrate (which could be replaced by ammonium formate or ammonium sulfate), 22% (w/v) PEG 3350 for *Sp*GAPDH, and 0.2 M sodium acetate, 20% (w/v) PEG 3350 for *Cp*GAPDH. Crystals were then cryoprotected with 20% (v/v) sterile glycerol, mounted in standard MicroMount (MiTeGen) and flash frozen in liquid nitrogen.

### Data Collection and Processing

Diffraction data were collected from flash-frozen crystals at 100 *K*. Data for *Sp*GAPDH crystals were collected on an ADSC CCD detector at the ID232 beamline (ESRF, Grenoble, France) (Flot et al., 2010) to a resolution of 1.5 Å, and *Cp*GAPDH crystals were collected using a photon-counting Pilatus 6M (DECTRIS Ltd., Baden, Switzerland) at the BL13-XALOC beamline (ALBA, Barcelona, Spain) (Juanhuix et al., 2014) to 2.55 Å resolution. Complete data sets were then processed with XDS (Kabsch, 2010a,b) and scaled and merged with Aimless (Evans and Murshudov, 2013) from the CCP4 software suite (Winn et al., 2011). Data collection and processing statistics are summarized in Table 1.

**Table 1:** Crystallographic data collection and refinement statistics.

	*Sp*GAPDH	*Cp*GAPDH
**Data collection**		
Wavelength (Å)	0.8726	0.9795
Space group	*P* 2_1_ 2_1_ 2_1_	*P* 2_1_
**Cell dimensions**		
*a, b, c* (Å)	79.27, 91.60, 106.27	73.28, 101.61, 92.82
α, β, γ (°)	90, 90, 90	90, 107.07, 90
Resolution range (Å)	44.13–1.50 (1.55–1.50)	44.37–2.55 (2.64–2.55)
Total no. of reflections	423,801 (22,988)	177,859 (17,860)
No. of unique reflections	122,574 (10,880)	42,338 (4,183)
Completeness (%)	98–53 (88.69)	99.61 (99.71)
Redundancy	3.5 (2.1)	4.2 (4.3)
⟨*I*⟩/σ(⟨*I*⟩)	15.81 (1.66)^#^	12.66 (1.24)^#^
R_meas_^a^	0.0707 (0.642)	0.0825 (1.126)
CC1/2^b^	0.997 (0.527)	0.998 (0.604)
**Refinement and validation**		
Reflections used	122,305 (10,879)	42,320 (4,183)
Reflections for R_free_	3030 (269)	2115 (209)
R_work_^c^	0.1486 (0.284)	0.1640 (0.313)
R_free_^d^	0.1633 (0.322)	0.2140 (0.378)
No. residues (chains)	672 (2)	1326 (4)
**R.m.s. deviations**		
Bond lengths (Å)	0.008	0.010
Bond angles (°)	1.28	1.41
***B*-factors (Å^2^)**		
Protein	14.87	74.12
Ligands	17.09	78.38
Water	31.02	60.97
**Ramachandran plot**		
Favored (%)	96.7	96.7
Outliers (%)	0.0	0.0

*Values for the highest resolution shell are given in parentheses. ^#^The maximum resolution of the data set was chosen so that CC1/2 > 0.5 and the ⟨*I*⟩/σ(⟨*I*⟩) > 1.2. ^*a*^Rmeas = 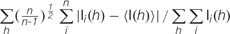, where n is the number of independent observations of *I*(**h**). ^*b*^ CC1/2 is the Pearson correlation coefficient calculated between two random half data sets. ^*c*^R_*work*_ = 
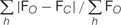
, where *F*_*O*_ and *F*_*C*_ are the observed and calculated structure factor amplitudes of reflection **h**. ^*d*^ R_*free*_ is as R_*work*_, but calculated with 5–10% of randomly chosen reflections omitted from refinement.*

### Model Building and Crystallographic Refinement

The structures of *Sp*GAPDH and *Cp*GAPDH were determined by the molecular replacement method using the program PHASER (McCoy et al., 2007) from the PHENIX program suite (Adams et al., 2010). A dimer of the *Staphylococcus aureus* GAPDH devoid of NAD+ (*Sa*GAPDH PDB ID 3LVF) (Mukherjee et al., 2010) was used as search model after mutation of the PDB file according to a sequence alignment (61% identity) with CHAINSAW (Stein, 2008). The fully refined structure of *Sp*GAPDH was then used as search model to solve *Cp*GAPDH crystal structure after sequence adjustment. Similar model building and refinement protocols were applied to solve the crystal structures of *Sp*GAPDH and *Cp*GAPDH. An omit map calculated from the model phases before (rigid) refinement or model building showed electron density corresponding to four NAD+ molecules, one per protomer. The complete homotetramer was then used for rigid-body and maximum likelihood refinement within phenix.refine (Afonine et al., 2012) setting aside 2.5% (*Sp*GAPDH) or 5.0% (*Cp*GAPDH) of the reflections (selected randomly) to create a data set of test reflections for cross-validation of the refinement procedure. Refinement cycles were interspersed with cycles of manual building (first, placing NAD+ and then solvent molecules) and validation with Coot (Emsley et al., 2010). Torsion-angle non-crystallographic symmetry restraints were applied during the initial refinement but were removed during the final refinement stages. Crystallographic refinement statistics are summarized in Table 1.

The coordinates and structure factors have been deposited in the Protein Data Bank (PDB) with accession codes 6FZH (*Sp*GAPDH) and 6FZI (*Cp*GAPDH). Authors will release the atomic coordinates and experimental data upon article publication.

## Results and Discussion

### Crystal Structures of *Sp*GAPDH and *Cp*GAPDH

**FIGURE 1:**
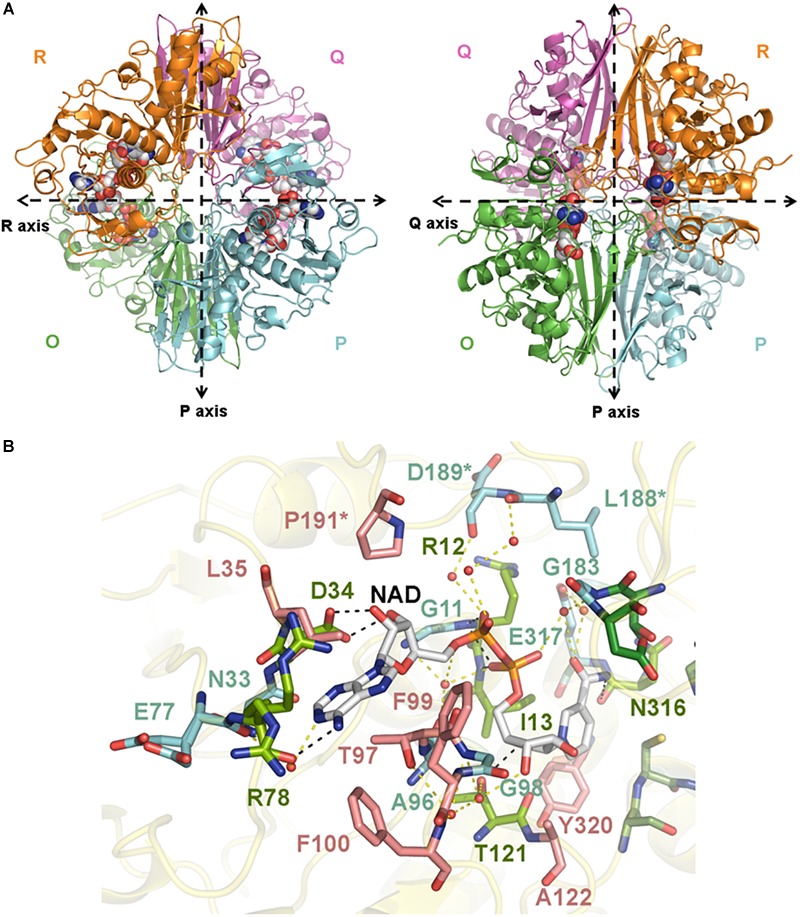
Crystal structure of *Sp*GAPDH. **(A)** Overall structure of *Sp*GAPDH (PDB 6FZH) in cartoon representation with chain colors (*O* in green, *P* in cyan, *Q* in violet, and *R* in orange). Dashed lines indicate the directions of the two molecular symmetry axes that lie on the plane of the figure. The NAD+ is shown in spacefilling representation and in CPK colors. Two views of the *Sp*GAPDH homotetramer are shown, with either the *Q*-axis or the *R*-axis oriented along an axis perpendicular to the paper. **(B)** NAD+-binding pocket. The NAD+ cofactor is shown in stick representation (carbon atoms in gray, other atoms in CPK colors). Residues that interact with NAD+ via direct hydrogen bonds are colored lime green and connected by black dashed lines; those residues that make indirect contacts with the cofactor via water-mediated hydrogen bonds are colored cyan and are connected by yellow dashed lines. Residues that interact with NAD+ through van der Waals interactions are shown in salmon. Cofactor-binding residues donated by the *S* loop of the adjacent subunit.

**FIGURE 2:**
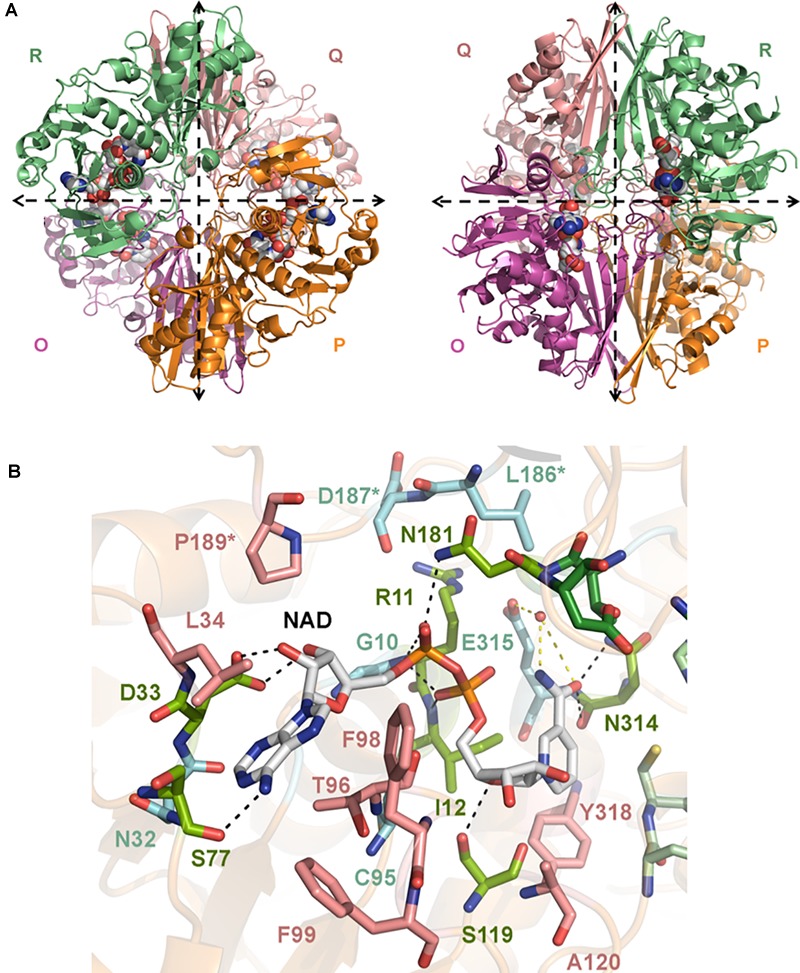
Crystal structure of *Cp*GAPDH. **(A)** Overall structure of *Cp*GAPDH (PDB 6FZI) in cartoon representation with chain colors (*O* in violet, *P* in orange, *Q* in salmon, and *R* in green). Dashed lines indicate the directions of the two molecular symmetry axes that lie on the plane of the figure. The NAD+ is shown in spacefilling representation and in CPK colors. Two views of the *Cp*GAPDH homotetramer are shown, with either the *Q*-axis or the *R*-axis oriented along an axis perpendicular to the paper. **(B)** NAD+-binding pocket. The NAD+ cofactor is shown in stick representation (carbon atoms in grey, other atoms in CPK colors). Residues that interact with NAD+ via direct hydrogen bonds are colored lime green and connected by black dashed lines; those residues that make indirect contacts with the cofactor via water-mediated hydrogen bonds are colored cyan and are connected by yellow dashed lines. Residues that interact with NAD+ through van der Waals interactions are shown in salmon. Cofactor-binding residues donated by the *S* loop of the adjacent subunit.

We have determined the first crystal structures of *Sp*GAPDH at 1.50 Å resolution (Figure 1) and *Cp*GAPDH at 2.55 Å resolution (Figure 2). Both crystal structures correspond to the holoenzymes with the NAD+ cofactor deeply buried into the active site (Figure 1B, 2B). The two GAPDH enzymes are sequence and structural homologs to *A. vaginae* (*Av*) GAPDH, whose structure was recently published (Querol-García et al., 2017). We solved them by molecular replacement using *Staphylococcus aureus* GAPDH devoid of NAD+ as a model (*Sa*GAPDH; PDB ID 3LVF) (Mukherjee et al., 2010). Crystallographic data processing and refinement and validation statistics are reported in Table 1.

The global architecture of *Cp*GAPDH and *Sp*GAPDH consists in a D2 homotetramer with *O, P, Q*, and *R* subunits, with three non-equivalent interfaces related by three mutually perpendicular axes (*P, Q*, and *R*) (Figure 1A, 2A). The N-terminal NAD+ (or cofactor) binding domain is an α/β/α Rossmann fold spanning residues 1–149 in *Cp*GAPDH and 1–151 in *Sp*GAPDH, which usually contains a central 7-stranded parallel β-sheet. Consecutive β-sheets in the cofactor binding domain are connected via short α-helices. The C-terminal catalytic domain spans residues 150–332 in *Cp*GAPDH and 152–336 in *Sp*GAPDH. The structure of the catalytic domain contains an 8-stranded parallel β-sheet with several α-helices and 3_10_ helices packed on both sides of the β-sheet. The two domains are connected at the catalytic cysteine residue (Cys150 in *Cp*GAPDH and Cys152 in *Sp*GAPDH), which is located between the last β-strand in the cofactor binding domain and the first α-helix of the catalytic domain. The p*K*a-lowering catalytic triad in GAPDH is completed by two absolutely conserved residues that can be identified by sequence comparisons and/or structure superposition, His177 and Arg233 in *Cp*GAPDH and His179 and Arg235 in *Sp*GAPDH. The conformation adopted by Arg233 in *Cp*GAPDH allows it to establish interactions with the nearby Thr180, Asp182, and Gln183 residues. Likewise, for *Sp*GAPDH, Arg235 interacts with Thr182, Asp184, and Gln185.

Although the so-called P_i_ and P_s_ sites cannot be visualized directly in our crystal structures because they were not co-crystallized with phosphate anions (or sulfate anions), they can be defined on the basis of a *S. aureus* (*Sa*) GAPDH structure crystallized with inorganic phosphate and NAD+ (PDB ID 3K73). The P_s_ site in *Cp*GAPDH (*Sp*GAPDH), which defines the place where the phosphate moiety of G3P is located, is formed by Thr180 (Thr182) Oγ, the carboxylate of Asp182 (Asp184), the guanidinium group of Arg233 (Arg235) and the 2′ hydroxyl of the nicotinamide-ribose (O2D) of NAD+. The P_i_ holding the inorganic phosphate substrate is instead formed by Thr210 (Thr212) and Gly211 (Gly213) plus the side chains of Ser149 (Ser151), His177 (His179) and Arg233 (Arg235), beside the main and side chains of Thr151 (Thr153).

The last important feature of GAPDH active site is the *S* loop, a long, winding segment of GAPDH that is found roughly midway between the cofactor-binding sites of its same subunit and the neighboring subunit. In *Cp*GAPDH (*Sp*GAPDH), the *S* loop comprises residues Ala178-Ile205 (Ala180-Ile207). The respective *S* loops provide part of the bridging region between the catalytic His177 and Arg233, and they contain residues Leu186 (Leu188), Asp187 (Asp189), and Pro189 (Pro190), which interact with the neighboring subunit related through rotation around the molecular *R* axis. Furthermore, Pro189 (Pro190) is part of the adenine-ribose binding pocket.

The C-terminal helix of the catalytic domain docks into a complementary groove in the N-terminal domain, thereby creating an extensive interdomain interface that stabilizes the bilobal structure of GAPDH and also contributes to the binding pocket for the nicotinamide ring of the NAD+ cofactor. The amino acids contributing to the NAD+ binding pocket of *Cp*GAPDH (*Sp*GAPDH) include direct hydrogen-bonding residues like Arg11 (Arg12), Ile12 (Ile13), and Ser77 (Arg78 and Asn316) (main-chain) and Asp33 (Asp34), Ser119 (Thr121) and Asn181 (side-chain), and Asn314 (both main-chain and side-chain), residues that interact through water-mediated hydrogen bonds, main chains of Gly10 (Gly11, Glu77, Ala96, Gly98, and Gly183) and Cys95 and side chains of Asn32 (Asn33) and Glu315 of the same monomer, plus Asp187 (Leu188 and Asp189) from the *S* loop of the adjacent monomer. The NAD+ binding pocket is completed by an array of hydrophobic residues from the same subunit (Leu34, Thr96, Phe98, Phe99, Ala120, and Tyr318 in *Cp*GAPDH, or Leu35, Thr97, Phe99, Phe100, Ala122, and Tyr320 in *Sp*GAPDH) and from the *S* loop of the adjacent monomer (Pro189 in *Cp*GAPDH or Pro191 in *Sp*GAPDH).

### Structural Homology Between GAPDH From Pathogenic Bacteria

**FIGURE 3:**
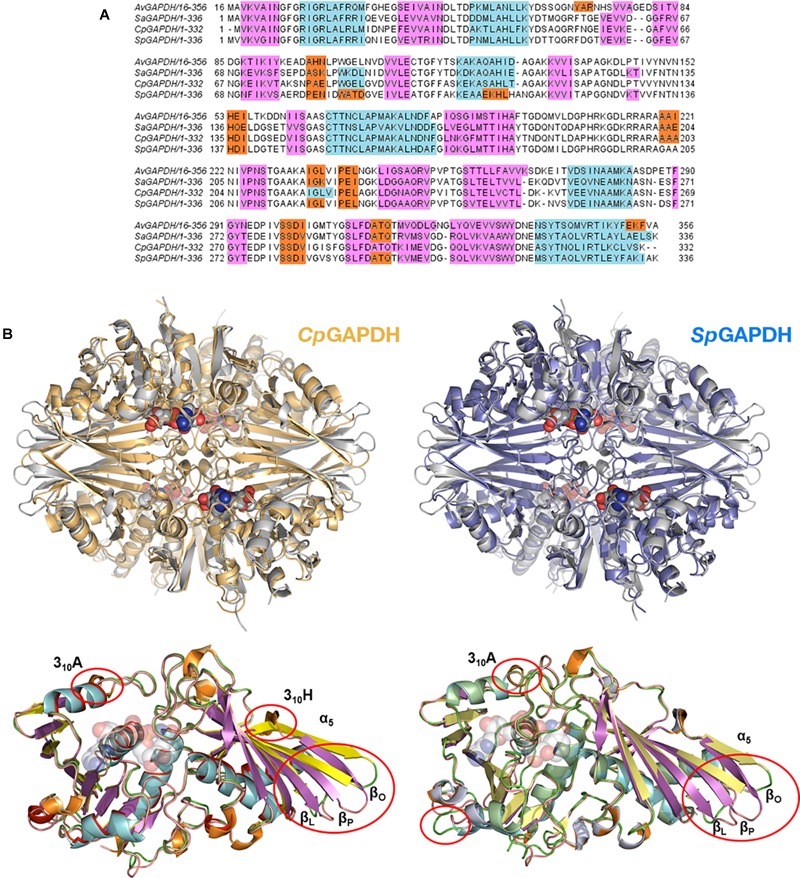
Structural homology. **(A)** Multiple sequence alignment of bacterial GAPDH sequences. Sequences are colored according to their secondary structure: α-helices in light blue, 3_10_ helices in orange and β-sheets in pink. **(B)** Superposition of *Cp*GAPDH (PDB 6FZI) (tetramer colored in wheat) and *Sp*GAPDH (PDB 6FZH) (blue) with *Av*GAPDH (PDB 5LD5) (gray). The largest differences are indicated by red ovals in the superposition between the corresponding monomeric structures.

The three enzymes analyzed here (*Cp*GAPDH, *Sp*GAPDH, and *Av*GAPDH) have pairwise sequence identities greater than 60% (Figure 3A) and, correspondingly, the root-mean-square differences (r.m.s.d.) upon superposition of the Cα atoms of the complete homotetramers vary from 1.3 to 1.4 Å. The r.m.s.d. values reduce to about 1.0 Å when only monomers are superimposed. Most differences cluster around connecting regions and loops on the surface of GAPDH (Figure 3B), although more specific structural differences can be noted between each specific pair of GAPDH enzymes at the *S* loops and in the vicinity of 3_10_ helices, as well as more limited discrepancies in the secondary structure adopted by short sequence segments.

**FIGURE 4:**
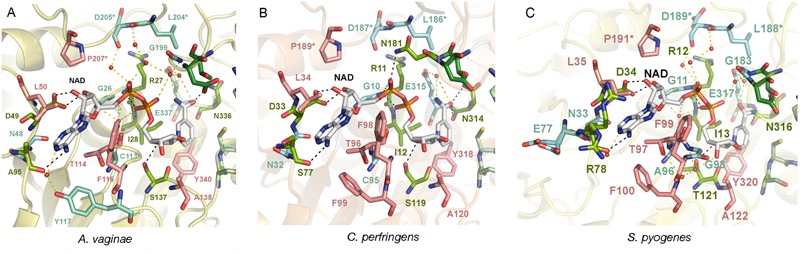
Adenine pocket. Close-up on the adenine-binding pockets of GAPDH from *A. vaginae, C. perfringens*, and *S. pyogenes*. The NAD+ cofactor and surrounding amino acid residues are shown in sticks. Residues that interact with the cofactor via direct hydrogen bonds are colored in lime green (with black dotted lines representing the bond) and those that are mediated by water molecules are colored in aquamarine (with yellow dotted lines as bonds). Residues making hydrophobic contacts with NAD+ are in pink. An asterisk (^∗^) denotes residues belonging to the adjacent *S* loop. **(A)** *Av*GAPDH (PDB 5LD5). **(B)** *Cp*GAPDH (PDB 6FZI). **(C)** *Sp*GAPDH (6FZH).

More important are the differences that occur at the active site, in particular those found in the adenine and nicotinamide binding subsites. In *Av*GAPDH, two changes occur with respect to *Sa*GAPDH around the adenine-binding subsite and the *S* loop, respectively (Querol-García et al., 2017). Ala95 replaces a Pro residue, and Leu204 replaces a Gln residue (Figure 4A). Since the interactions contributed by Pro and the water-mediated hydrogen bond by Gln only involve main-chain atoms, the strength of NAD+ binding is essentially unaltered while the unique features provided for by Ala95 and Leu204 remain accessible for drug differential binding.

When *Av*GAPDH and *Cp*GAPDH are compared (Figure 4B), differences around the adenine binding pocket become evident. One difference is found in the loop spanning residues 93–97 of *Av*GAPDH, where Ala95 is substituted for Ser in *Cp*GAPDH. Since the relevant interaction is water-mediated, the substitution does not affect the main-chain carbonyl hydrogen bond. Another change concerns the substitution of Tyr117 (*Av*GAPDH) by Phe in *Cp*GAPDH, thereby losing the water-mediated hydrogen bond made between Tyr117 hydroxyl with the N1 and N6 atoms of adenine. Finally, the last difference between *Av*GAPDH and *Cp*GAPDH in the NAD+ binding pocket is the substitution of Gly199 (*Av*GAPDH) by Asn181 (*Cp*GAPDH). The net effect of this substitution is that the interaction between Leu186 from the *S* loop of a neighboring subunit with the opposite NAD+ cofactor occurs via Asn181 in *Cp*GAPDH rather than through a water molecule in *Av*GAPDH.

Comparing the adenine-binding subsites of *Av*GAPDH with *Sp*GAPDH, there are two important substitutions (Figure 4C): Ala95 to Arg (although the interaction with NAD+ occurs through main-chain hydrogen bonds) and, analogously to *Cp*GAPDH, Tyr117 is a Phe in *Sp*GAPDH.

**FIGURE 5:**
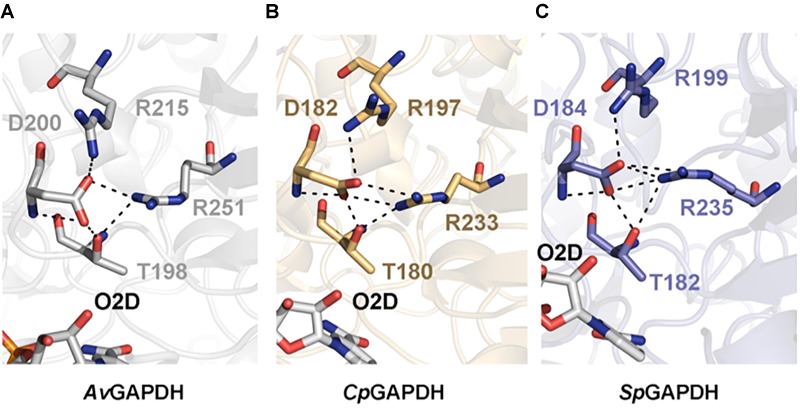
Substrate phosphate sites. Residues that contribute to the P_s_ are shown in sticks and in chain color. In all cases, NAD+ is shown in CPK colors. **(A)** *Av*GAPDH (PDB 5LD5). **(B)** *Cp*GAPDH (PDB 6FZI). **(C)** *Sp*GAPDH (PDB 6FZH).

The phosphate binding sites (P_i_ and P_s_) are very conserved among the GAPDH enzymes examined. In particular, the P_s_ sites (Figure 5) deviate only from the consensus in that residues 182, 184, and 200 of *Av*GAPDH, *Cp*GAPDH, and *Sp*GAPDH, respectively, are negatively charged Asp residues instead of a Ser/Thr residue as present in other GAPDH enzymes. To stabilize the phosphate moiety from the substrate at P_s_, these enzymes have an Arg residue at position 215, 197, and 199 of *Av*GAPDH, *Cp*GAPDH, and *Sp*GAPDH, respectively, which also interact with the previous Asp residue. Other bacterial GAPDH enzymes like *B. stearothermophilus* and *S. aureus* also share a similar mechanism for the stabilization of the substrate phosphate group, lending support to the notion that the tandem Asp–Arg substitute for the single Ser/Thr residue observed in human and protozoan GAPDH enzymes.

The inorganic phosphate subsite, Pi, is also largely conserved across all the analyzed bacterial GAPDH enzymes. The controversy about the existence of two types of P_i_ sites, a “classic” (Skarzyński et al., 1987) and a “new” P_i_ (Korndörfer et al., 1995) site, cannot be resolved with our structures since they were not crystallized with either phosphate or sulfate, nor do they contain covalent intermediates bound to the catalytic Cys residue. The structure of *Sa*GAPDH crystallized with phosphate anions but without NAD+ (PDB ID 3L6O) or with a covalently bound thioacylated intermediate (PDB ID 3LC2) (Mukherjee et al., 2010) exhibit a “new” P_i_ site, whereas other *Sa*GAPDH structures show more “classic” P_i_ sites. The movement of the active-site segment 209–215 (in *Sa*GAPDH numbering) toward the front would define the “new” P_i_ site configuration. Therefore, we can safely assume, giving the sequence and structural homology, that *Av*GAPDH, *Cp*GAPDH, and *Sp*GAPDH should also experience a conformational change leading to the formation of “new” P_i_ sites under the same conditions where a “new” P_i_ site is observed in *Sa*GAPDH.

**FIGURE 6:**
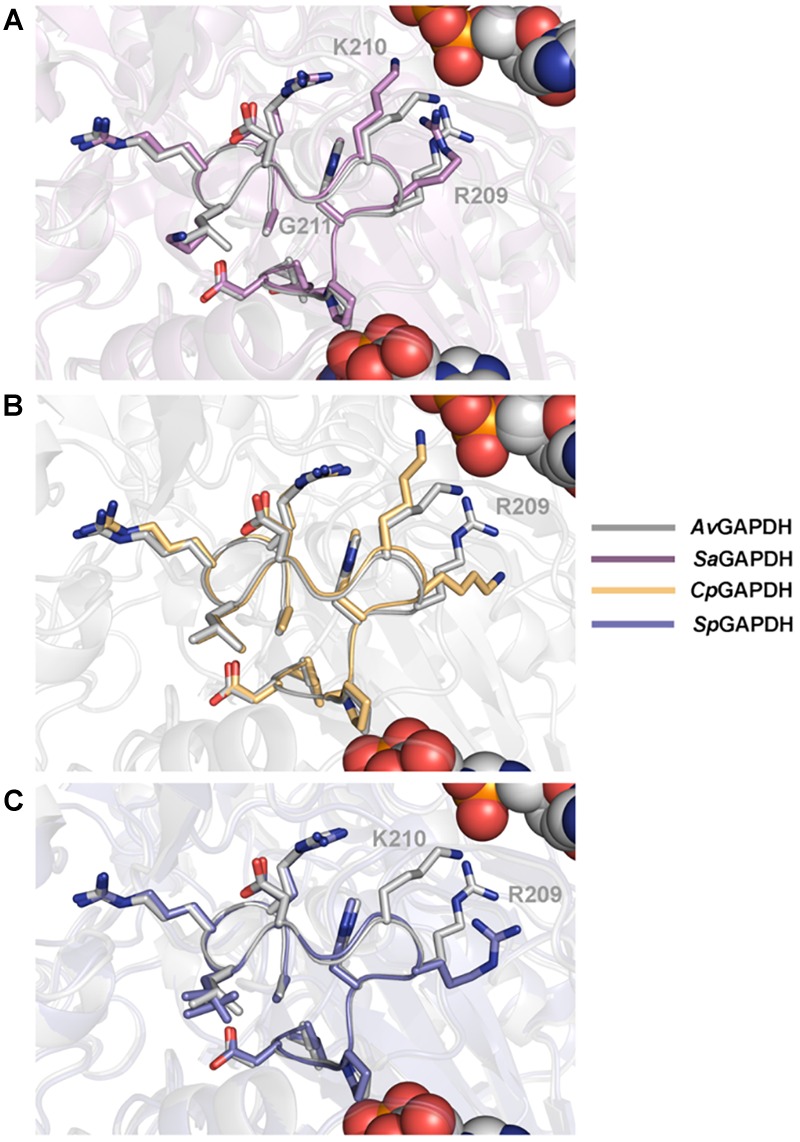
Structural homology between *S* loops. Pairwise structural superposition of the *S* loop of *Av*GAPDH (PDB 5LD5) (gray) with **(A)** *Sa*GAPDH (PDB 3K73) (violet), **(B)** *Cp*GAPDH (PDB 6FZI) (orange), and **(C)** *Sp*GAPDH (PDB 6FZH) (blue). The side chains of residues 209–217 of the *S* loop (*Av*GAPDH numbering) are depicted in sticks.

The structure of the *S* loop is essentially conserved across *Av*GAPDH, *Cp*GAPDH, and *Sp*GAPDH (Figure 6), thus contributing equally to stabilizing the interaction between neighboring subunits. The major difference is seen between *Av*GAPDH (and *Sa*GAPDH) and *Cp*GAPDH/*Sp*GAPDH. Whereas in the *S* loop of *Av*GAPDH (209–217), Arg209, and Lys210 stick out toward the NAD+ bound in the same subunit, in *Cp*GAPDH a Lys residue substitutes Arg209 of *Av*GAPDH that points toward the protein–protein interface rather than toward the NAD+ cofactor. Furthermore, in *Sp*GAPDH a Gly residue substitutes Lys210 of *Av*GAPDH, completely eliminating the positive charge.

### Comparison With Human Liver GAPDH

Comparison with the structure of human liver GAPDH (*Hs*GAPDH) (PDB ID 1ZNQ) (Ismail and Park, 2005) is informative because structural and chemical features that are distinctive between the GAPDH of bacterial pathogens and *Hs*GAPDH can be exploited for the discovery of selective inhibitors. Selective inhibitors avoiding the first pass metabolism in the liver have in principle two advantages: (1) their concentrations in systemic circulation will probably be greater, and (2) less adverse effects by not interfering with liver *Hs*GAPDH’s physiological function.

**FIGURE 7:**
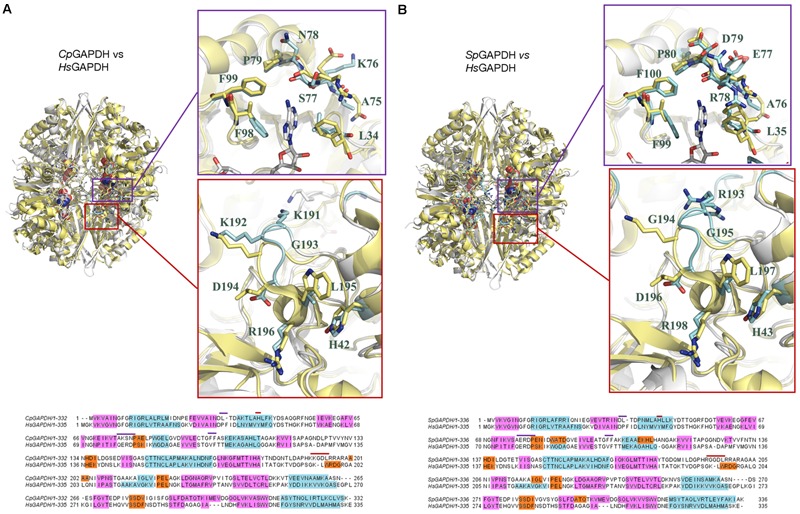
Comparison of bacterial and human liver GAPDH active site structure. **(A)** Comparison with *Cp*GAPDH (PDB 6FZI). **(B)** Comparison with *Sp*GAPDH (PDB 6FZH). In both cases, *Hs*GAPDH (PDB 1ZNQ) is shown in yellow color. NAD+ molecules are depicted as spacefilling models. Regions where structural differences are more noticeable are boxed with a blue outline (adenine pocket) or a red outline (*S* loop). A pairwise sequence alignment is shown underneath the corresponding superimposed structures. Structural elements are color coded (α-helices in cyan, 3_10_ helices in orange and β-sheets in cyan). Residues in the alignment that contribute to the adenine-binding pocket or to the *S* loop are denoted by blue or red segments, respectively.

Critical active site residues in the adenine-binding pocket are poorly conserved between *Cp*GAPDH (^75^AKSN^78^) and *Hs*GAPDH (^78^QERD^81^) (Figure 7A) while nearly perfectly conserved in *Sp*GAPDH (^76^AERD^79^) (Figure 7B). The loss of conservation in *Cp*GAPDH might have functional consequences. Besides the overall alteration in charge distribution around the adenine pocket, the most significant change appears to be the rejection of a positive side chain in *Cp*GAPDH from the inside face of the adenine pocket. In contrast, in *Sp*GAPDH and *Hs*GAPDH, an arginine side chain seems firmly inserted into the adenine pocket. This additional positive charge may contribute to the overall binding energy for NAD+. As for the orientation of the adenine ring, we found an equivalent pair of aliphatic/aromatic residues in *Cp*GAPDH/*Sp*GAPDH and *Hs*GAPDH: Leu34/Leu35 and Phe98/Phe99 for Phe37 and Val101 in *Hs*GAPDH. Those interactions participate in anchoring the NAD+ while keeping the orientation of the adenine ring. Taking into consideration that many known GAPDH inhibitors target the adenine subpocket, the non-conservative changes observed in *Cp*GAPDH may have consequences for the binding strength and selectivity of GAPDH-targeted drugs.

The *S* loop also contains several positively charged residues in *Cp*GAPDH and *Sp*GAPDH that are different, neutral or negatively charged in *Hs*GAPDH. For example, Lys191 and Arg197 (*Cp*GAPDH) correspond, respectively, to Gly193 and Asp198 (*Hs*GAPDH). Similarly, Arg193 and Gly194 (*Sp*GAPDH) correspond to Gly193 and Lys194 (*Hs*GAPDH). In addition, insertions into the *S* loop of *Cp*GAPDH causes Lys191 side chain to point toward the opposite neighbor surface, and the same effect occurs for Arg199 in *Sp*GAPDH (instead of Asp198 in *Hs*GAPDH). These observations suggest that the *S* loop may represent a second site for targeting inhibitors with selectivity toward the bacterial enzymes. Depending on the amount and distribution of negative charge on the inhibitor, it may be possible to further optimize inhibitor binding against *Cp*GAPDH (targeting the Lys191/Lys192 dipeptide) (Figure 7A) or *Sp*GAPDH (focusing on Arg193) (Figure 7B).

### Enzyme Kinetics

We measured the steady-state kinetic parameters for *Sp*GADPH and *Cp*GAPDH with respect to the three substrates (NAD+, G3P, and P_i_) by varying the concentration of each substrate at a time while maintaining fixed concentrations of the other two substrates. The complete set of kinetic parameters are shown in Table 2, along with those of *A. vaginae* GAPDH (Querol-García et al., 2017). The three GAPDH enzymes exhibited roughly similar values for the kinetic parameters except for *k*_cat_ (and *k*_cat_/*K*_M_), which was significantly greater for *Sp*GAPDH. In contrast to *Av*GAPDH and *Sp*GAPDH, *Cp*GAPDH exhibited cooperative behavior for all substrates. For *Cp*GAPDH, fitting of the corresponding initial velocity curves to a Hill model yielded an approximate value for the Hill constant of 2.0.

**Table 2:** GAPDH enzyme kinetic parameters.

Enzyme	Substrate	Kinetic model	*K*_M_ (mM)	*V*_max_ (mM s^-1^ mg^-1^)	*k*_cat_ (s^-1^)	*k*_cat_/*K*_M_ (mM s^-1^)
*Sp*GAPDH	G3P	Michaelis	2.8 ± 0.3	61.1 ± 2.6	6110 ± 260	2182 ± 320
	NAD+	Michaelis	0.28 ± 0.03	37.3 ± 1.1	3730 ± 100	13,321 ± 1800
	P_i_	Michaelis	2.9 ± 0.2	34.5 ± 0.8	3450 ± 80	1189 ± 109
*Cp*GAPDH	G3P	Hill (*n* = 2.1 ± 0.3)	2.8 ± 0.2	10.6 ± 0.4	107 ± 4	38 ± 4
	NAD+	Hill (*n* = 2.0 ± 0.2)	0.18 ± 0.01	6.8 ± 0.1	68 ± 1	378 ± 29
	P_i_	Hill (*n* = 1.6 ± 0.1)	8.4 ± 0.4	8.7 ± 0.2	87 ± 4	10 ± 1
*Av*GAPDH^a^	G3P	Michaelis	2.6 ± 0.5	5.7 ± 0.3	57 ± 6	22 ± 5
	NAD+	Michaelis	0.08 ± 0.03	3.1 ± 0.2	31 ± 3	348 ± 150
	P_i_	Michaelis	3.4 ± 0.6	2.9 ± 0.2	29 ± 3	8 ± 2

*^*a*^Kinetic parameters for *Av*GAPDH were taken from Querol-García et al. (2017).*

### Inhibition by Anacardic Acid and Curcumin

**FIGURE 8:**
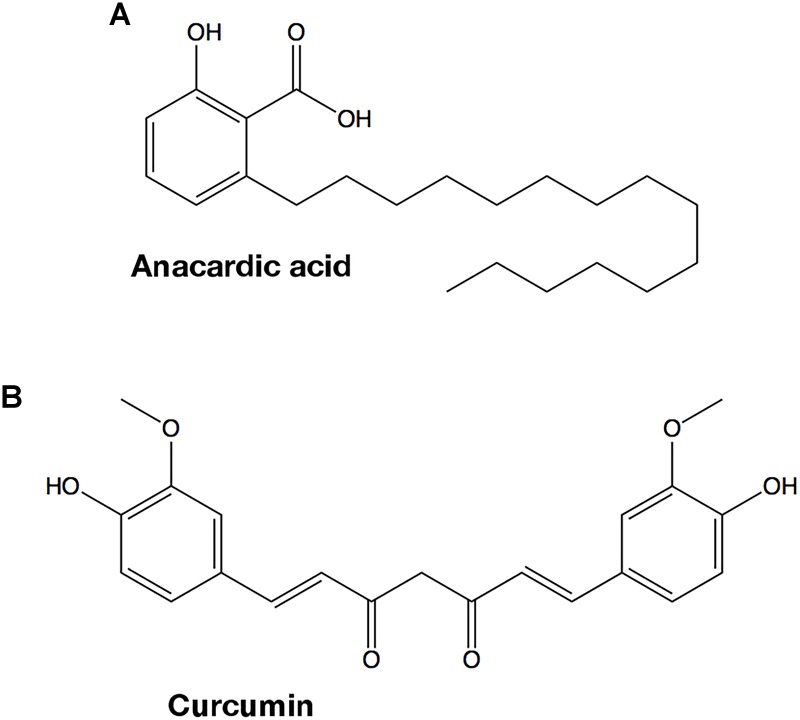
Chemical structures of GAPDH inhibitors. **(A)** Anacardic acid. **(B)** Curcumin.

Natural products contain active principles with biological functions, including antimicrobial and antibiotic properties, which can be used to treat infections. The recent discovery that both anacardic acid and curcumin, two small molecules that are isolated from plants used in human nutrition, have potent antibacterial activities when administered to cell cultures, prompted us to evaluate whether GAPDH could be one of the intracellular targets of those compounds. Anacardic acid (6-pentadecylsalicylic acid) is purified from the oil of *Anacardium occidentale* (Tyman and Kiong, 1978) and consists in a salicylic acid moiety with a long unbranched side chain attached to position 6 of the phenol ring (Figure 8A). The second compound, curcumin (Figure 8B), is the principal polyphenolic phytochemical molecule found in the rhizome of *Curcuma longa* and its antibacterial properties have long been known (Schraufstatter and Bernt, 1949). Both compounds share two main traits. Firstly, they contain substituted phenol rings in their structures, one in anacardic acid and two in curcumin. Secondly, one of the ring substituents is a long linear chain of carbon atoms. In curcumin, this linear substituent is shorter than in anacardic acid but it ends in a second phenol moiety.

**FIGURE 9:**
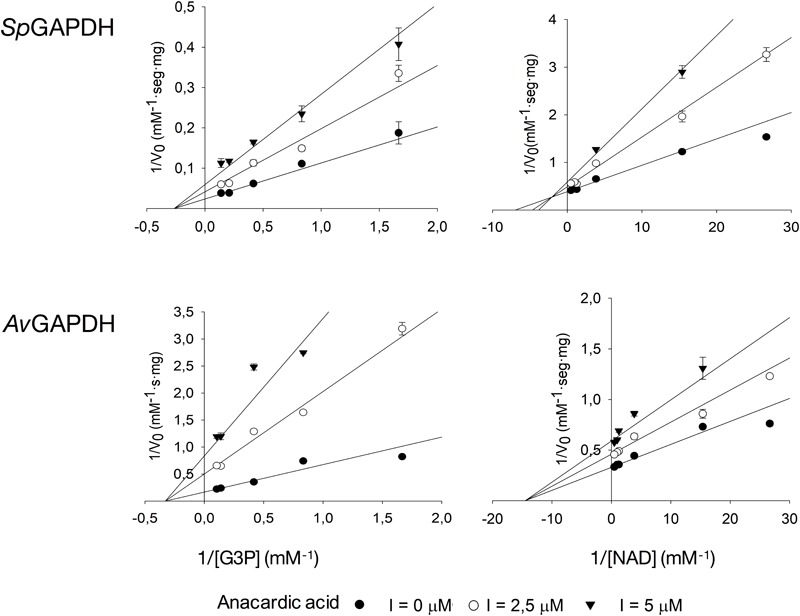
Inhibition of *Sp*GAPDH and *Av*GAPDH by anacardic acid. Effect of increasing concentrations of anacardic acid on the kinetic parameters of *Sp*GAPDH and *Av*GAPDH for G3P and NAD+ (*Cp*GAPDH was not affected). Double-reciprocal representation of the inhibition kinetics, which is diagnostic of the non-competitive model of inhibition.

**FIGURE 10:**
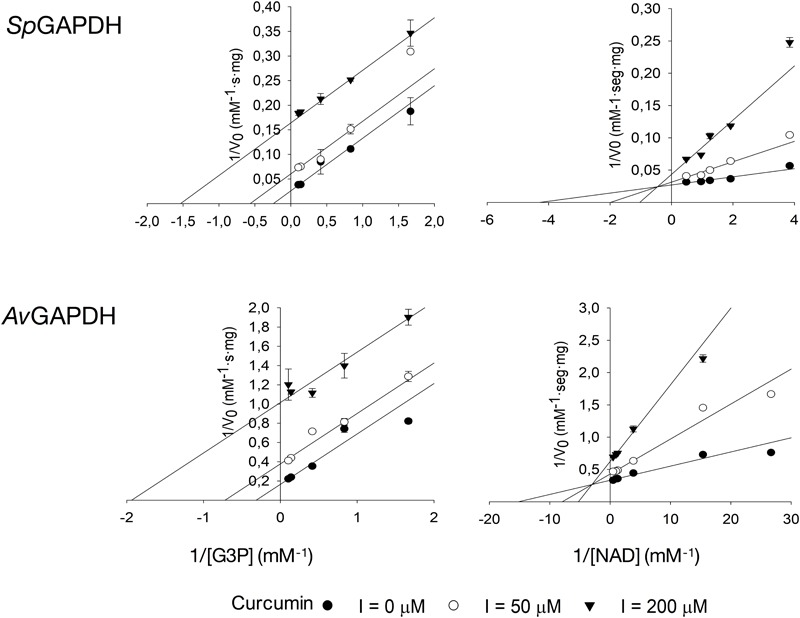
Inhibition of *Sp*GAPDH and *Av*GAPDH by curcumin. Effect of increasing concentrations of curcumin on the kinetic parameters of *Sp*GAPDH and *Av*GAPDH for G3P and NAD+ (*Cp*GAPDH was not affected). Double-reciprocal representation of the inhibition kinetics, which is diagnostic of the uncompetitive model of inhibition.

While anacardic acid has been previously shown to inhibit GAPDH from *T. cruzi* in a non-competitive fashion with respect to G3P and NAD+ (Pereira et al., 2008), there was no antecedent relating curcumin with GAPDH inhibition. To assess the potential inhibitory activity of both compounds on GAPDH from Gram-positive bacterial pathogens, we tested the effect of two concentrations of anacardic acid (2.5 and 5.0 μM) and curcumin (50 and 200 μM) on the steady-state kinetic parameters of *Sp*GAPDH, *Cp*GAPDH, and *Av*GAPDH. Despite the sequence and structural similarity and roughly similar kinetic parameters, *Cp*GAPDH was not affected by the concentrations used of either inhibitor. Table 3 lists the kinetic parameters obtained from the inhibition experiments (Figure 9, 10).

**Table 3:** Inhibition kinetics parameters.

Inhibitor	Enzyme	Substrate	Inhibition modality^a^	*K*_i_ (μM)
Anacardic acid	*Sp*GAPDH	G3P	NC	3.3 ± 0.3
		NAD+	NC	2.8 ± 0.9
	*Av*GAPDH	G3P	NC	1.25 ± 0.08
		NAD+	NC	6.3 ± 0.4
Curcumin	*Sp*GAPDH	G3P	UC	38 ± 4
		NAD+	NC	32 ± 7
	*Av*GAPDH	G3P	UC	39 ± 3
		NAD+	NC	28 ± 5

*^*a*^NC, non-competitive; UC, uncompetitive.*

Anacardic acid inhibited GAPDH through a non-competitive mechanism with respect to both G3P and NAD+ substrates. With nearly equal low micromolar *K*_i_ values for both substrates, anacardic acid is a potent GAPDH inhibitor. Its non-competitive inhibition mechanism allows anacardic acid to bind to both the free enzyme and the enzyme-substrate complex, presumably in a surface pocket little affected by substrate binding or catalytic turnover. Since the inhibition modality and *K*_i_ of anacardic acid was identical for *Sp*GAPDH and *Av*GAPDH, they are likely to share a similar anacardic acid-binding pocket. It is remarkable that *Cp*GAPDH could not be inhibited by anacardic acid at the concentrations tested despite the sequence and structural similarity with *Sp*GAPDH and *Av*GAPDH.

Curcumin, in contrast, is a 10-fold less potent inhibitor and it acts through two different inhibitor modalities depending on the substrate. While curcumin is an uncompetitive inhibitor with respect to G3P for *Sp*GAPDH and *Av*GAPDH, it behaves as a non-competitive inhibitor with respect to NAD+. Taken together, these observations indicate that curcumin binds either to the GAPDH-G3P complex or to an enzyme configuration created by G3P binding. The non-competitive inhibitor modality of curcumin with respect to NAD+ demonstrates that the cofactor is not involved in, nor does its presence affect, curcumin binding.

The different inhibition mechanisms in operation for anacardic acid and curcumin expose the difference in GAPDH binding modes between the two compounds. While anacardic acid most likely targets a preformed pocket on the enzyme’s surface that is accessible in both the free enzyme and the Michaelis complex (non-competitive inhibition with a single *K*_i_), curcumin seems to prefer a direct attachment to a GAPDH-G3P complex and consequently exhibits a pure uncompetitive inhibition modality.

The observed differences between GAPDH from various pathogenic bacteria with respect to their susceptibility to inhibition by anacardic acid and curcumin (e.g., *Cp*GAPDH cannot be inhibited by either of them), the inhibition potency (*K*_i_ for anacardic acid is 10-fold smaller than for curcumin for *Sp*GAPDH and *Av*GAPDH), and the inhibition modality (non-competitive versus uncompetitive), all reflect the underlying structural and chemical disparities between these otherwise highly conserved enzymes. Even in the absence of crystal structures for the GAPDH-inhibitor complexes, the differential behavior of *Sp*GAPDH, *Cp*GAPDH, and *Av*GAPDH to anacardic acid and curcumin, despite their considerable sequence and structural conservation, points to the existence of sufficient differences to afford selective inhibition through lead optimization efforts. Given the greater disparities that separate bacterial and human liver GAPDH, the identification of un- and non-competitive inhibitors and then the judicious exploitation of these inhibitor-binding pockets will open the avenue for the advent of more potent, highly selective bacterial GAPDH inhibitors.

### Anacardic Acid and Curcumin Inhibit Growth of *S. pyogenes*

Since anacardic acid and curcumin are both capable of inhibiting GAPDH, we decided to assay their effect when added directly to dividing cells of clinically relevant bacterial strains by using standard antimicrobial susceptibility tests. We chose serotype M1 group A *S. pyogenes* strains 950383, 941079, 950358, and 950771 previously characterized in our laboratories which had been isolated from clinical cases of necrotizing fasciitis (Pérez-Caballero et al., 2000). Of those, only strain 950383 had been observed to interact with human complement FHL-1 (Pérez-Caballero et al., 2000). Both anacardic acid and curcumin were indeed capable of halting microbial growth when used at sub-millimolar concentrations. Our antimicrobial susceptibility tests demonstrated that anacardic acid exhibited a greater antimicrobial activity than curcumin (Table 4). Thus, all four *S. pyogenes* strains were susceptible to low concentrations of anacardic acid (0.5–2 μg/ml, 1.4–5.7 μM) with minimal inhibitory concentration (MIC) of 1.4 μM (0.5 μg/ml), while at least 128 μg/ml of curcumin (347 μM) were required to inhibit bacterial growth. This result is in agreement with a recent report on the growth inhibitory properties of anacardic acid over *S. pyogenes* and *S. agalactiae* that reported MIC values of 5 μM (Hamad and Mubofu, 2015; Hollands et al., 2016). While anacardic acid was able to inhibit growth at concentrations close to its determined *K*_i_, the curcumin concentration necessary to elicit minimal growth inhibition was significantly greater than its *K*_i_ (38 vs. 347 μM). The latter discrepancy indicates that the biological activity of curcumin is reduced by one or possibly many factors including lower membrane permeability, off-target effects, and low-level metabolic turnover.

**Table 4:** Minimum inhibitory concentration (MIC) of curcumin and anacardic acid against evaluated strains.

	MIC anacardic acid		MIC curcumin	
Strains	(μg/ml)	(μM)	(μg/ml)	(μM)
*S. pyogenes* 950383	0.5	1.4	128	347
*S. pyogenes* 941079	0.5	1.4	128	347
*S. pyogenes* 950358	0.5	1.4	128	347
*S. pyogenes* 950771	2.0	5.7	128	347

### Interaction of GAPDH With Human Complement Factors

Besides the pivotal role that GAPDH plays in bacterial catabolism, GAPDH can also act as an effective virulence factor by targeting and sequestering human complement factors like C3, C1q, and the anaphylatoxin C5a (Terao et al., 2006; Terrasse et al., 2012; Querol-García et al., 2017). A prerequisite for bacterial GAPDH to display such complement immunoevasive functions is the colocalization of GAPDH and the targeted complement proteins. Colocalization can be achieved by exporting GAPDH to the extracellular side of the cell wall, where it can remain associated with cell-wall structures or be released into the medium. GAPDH export has already been demonstrated for various bacterial and eukaryotic organisms through a variety of molecular mechanisms (Terao et al., 2006; Matta et al., 2010; Aguilera et al., 2012; Seidler, 2013; Sahoo et al., 2013).

**FIGURE 11:**
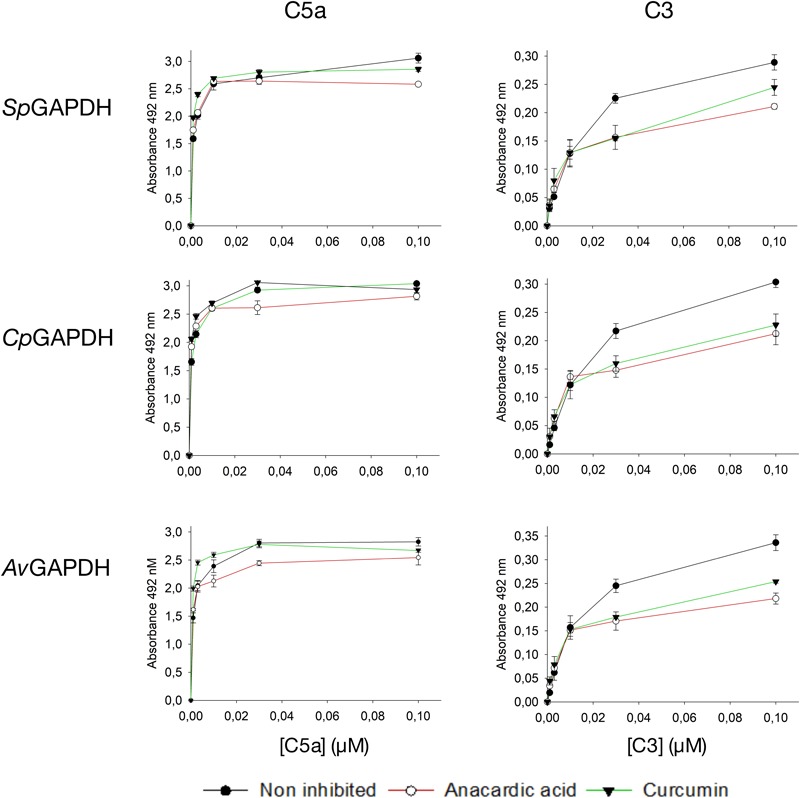
Interaction of human complement factors C5a, C3a, or C3 with surface-immobilized *Av*GAPDH, *Sp*GAPDH, and *Cp*GAPDH measured by an ELISA assay. Absorbance at 492 nm is plotted along the *y*-axis against increasing C5a, C3a, or C3 concentration on the *x*-axis. Data points and error bars represent mean ± SD (standard deviation; *N* = 3). Interaction with C5a **(A)** or C3 **(B)** in absence and at fixed concentration of anacardic acid (5 μM) or curcumin (50 μM).

Having previously analyzed the C5a binding activity of *Sp*GAPDH, *Cp*GAPDH, and *Av*GAPDH (Querol-García et al., 2017), we resorted to enzyme-linked immunosorbent assays (ELISA) to test whether GAPDH from these bacteria might be able to bind not only C5a but also the closely related anaphylatoxin C3a and its precursor C3. C3 was chosen because it has been reported to be a target for extracellular GAPDH from a parasitic nematode (Sahoo et al., 2013). C5a binding was assayed for *Sp*GAPDH and *Cp*GAPDH, extending the previously published results on *Av*GAPDH (Querol-García et al., 2017). All three GAPDH were capable of binding C5a and, albeit with lower affinity, also C3 in these assays in a dose-dependent manner (Figure 11). This is an important observation that is in line with recent observations concerning GAPDH from *S. pneumoniae* and from the parasite nematode *Haemonchus contortus*, both of which simultaneously target more than one complement factor (Terrasse et al., 2012; Sahoo et al., 2013; Vedamurthy et al., 2015). However, we could not detect binding of C3a to GAPDH above background level, indicating a high degree of selectivity of GAPDH for C5a (Figure 11).

Next, we tested whether anacardic acid and/or curcumin could prevent GAPDH binding to C5a or C3 using the same ELISA assay described above. Since the N-terminus of both *Sp*GAPDH and *Av*GAPDH has been implicated in C5a binding and this region includes the catalytic Cys residue and an extended loop that closes the active site cavity, we speculated whether changes in or near the active site cavity could disrupt C5a recognition. We also probed the effect that anacardic acid or curcumin might have on C3 binding, even though it is less likely that a small-molecule inhibitor could prevent GAPDH from binding to a far greater protein like C3. Indeed, the results show that the presence of either inhibitor at concentrations that achieve nearly full inhibition of GAPDH catalytic activity were not effective at disrupting the interaction with C5a and only poorly effective with C3 (Supplementary Figure S5). Taken together, these observations demonstrate that the surface areas that GAPDH uses to interact with C5a and C3 are either distinct (non-overlapping or only partially overlapping) or are not significantly altered by binding of anacardic acid or curcumin. These results point to the functional segregation between the C5a and C3 interaction surfaces and the GAPDH binding pockets for the two natural compounds in the assayed systems. Given that the moonlighting functions of GAPDH as an immune evasive factor take place in the extracellular space, they are likely decoupled from the enzymatic activity.

### Relevance of GAPDH-Targeting Antimicrobials

Bacterial infections are a significant medical and economic threat to human societies. Antibiotics have long been used to control bacterial infections successfully. However, their widespread use has had the undesired effect of promoting the emergence of multidrug resistant bacteria, which represent a serious danger for human health, especially for immunocompromised patients in the hospital environment. In this work, we have characterized the inhibitory potency and modality of two natural products, anacardic acid and curcumin, toward the ubiquitous and highly conserved glycolytic enzyme GAPDH from several Gram-positive bacterial pathogens. Owing to the vital role it plays in secondary metabolism, GAPDH may be an interesting target for drug discovery provided that inhibitors can be proved safe, efficacious and selective toward the bacterial enzymes. Low micromolar concentrations of anacardic acid and, to a lesser degree, curcumin can inhibit growth of clinically isolated bacterial cultures and inhibit GAPDH *in vitro*, thereby suggesting that GAPDH might be the relevant physiological target for their antimicrobial activities. Since pathogenic bacterial GAPDH can also interfere with the human C5a anaphylatoxin-mediated signaling, it is important to understand whether the antimicrobial activity of these compounds could also interfere with GAPDH’s immune evasion activity. Our results indicate that these two functions are unrelated. Deciphering the high-resolution structure of *S. pyogenes* and *C. perfringens* GAPDH will pave the way for the discovery of selective inhibitors based on anacardic acid and curcumin.

## Author Contributions

MV, FF, SA, and SRC conceived the experimental study, designed the experiments, analyzed the data, and wrote the manuscript. JQ-G and SG expressed and purified *Sp*GAPDH, *Cp*GAPDH, *Av*GAPDH, and C5a, and GS-B expressed and purified C3a. VF-H helped with the initial expression and purification of *Sp*GAPDH and *Cp*GAPDH. JQ-G, FF, and MV crystallized *Sp*GAPDH and *Cp*GAPDH. FF and MV collected the X-ray data set from both crystals, determined, and analyzed the crystal structures. SG measured enzyme kinetics and the inhibition by anacardic acid and curcumin. MS and SRC provided anti-C5a, anti-C3a, and anti-C3 antibodies for ELISA assays. SG and GS-B performed the ELISA assays. ÀG-A and SA measured the MIC of anacardic acid and curcumin with clinical isolates of *S. pyogenes*. SA provided materials and helped design the experiments and analyzed the data. All authors contributed critically to the manuscript.

## Conflict of Interest Statement

The authors declare that the research was conducted in the absence of any commercial or financial relationships that could be construed as a potential conflict of interest.
