# Supplementary material for: The Antimicrobials Anacardic Acid and Curcumin Are Not-Competitive Inhibitors of Gram-Positive Bacterial Pathogenic Glyceraldehyde-3-Phosphate Dehydrogenase by a Mechanism Unrelated to Human C5a Anaphylatoxin Binding
Source: Front Microbiol. 2019 Feb 26;10:326. doi: 10.3389/fmicb.2019.00326 (PMC6400076; doi:10.3389/fmicb.2019.00326)
Supplement: Supplementary file 1 [file Data_Sheet_1.PDF]

Supplementary Material

The Antimicrobials Anacardic Acid and Curcumin are Not-Competitive Inhibitors of Gram-positive Bacterial Pathogenic Glyceraldehyde-3-phosphate Dehydrogenase by a Mechanism Unrelated to Human C5a Anaphylatoxin Binding

Sara Gómez[1], Javier Querol-García[1,4], Gara Sánchez-Barrón[1,5], Marta Subias[1,2], Àlex González-Alsina[3], Virginia Franco-Hidalgo[1], Sebastián Albertí[3], Santiago Rodríguez de Córdoba[1,2], Francisco J. Fernández[1,5,*], M. Cristina Vega[1,*]

*** Correspondence:** Corresponding authors: fjfernandez@abvance.com; cvega@cib.csic.es; cristina.vega@strubicib.org

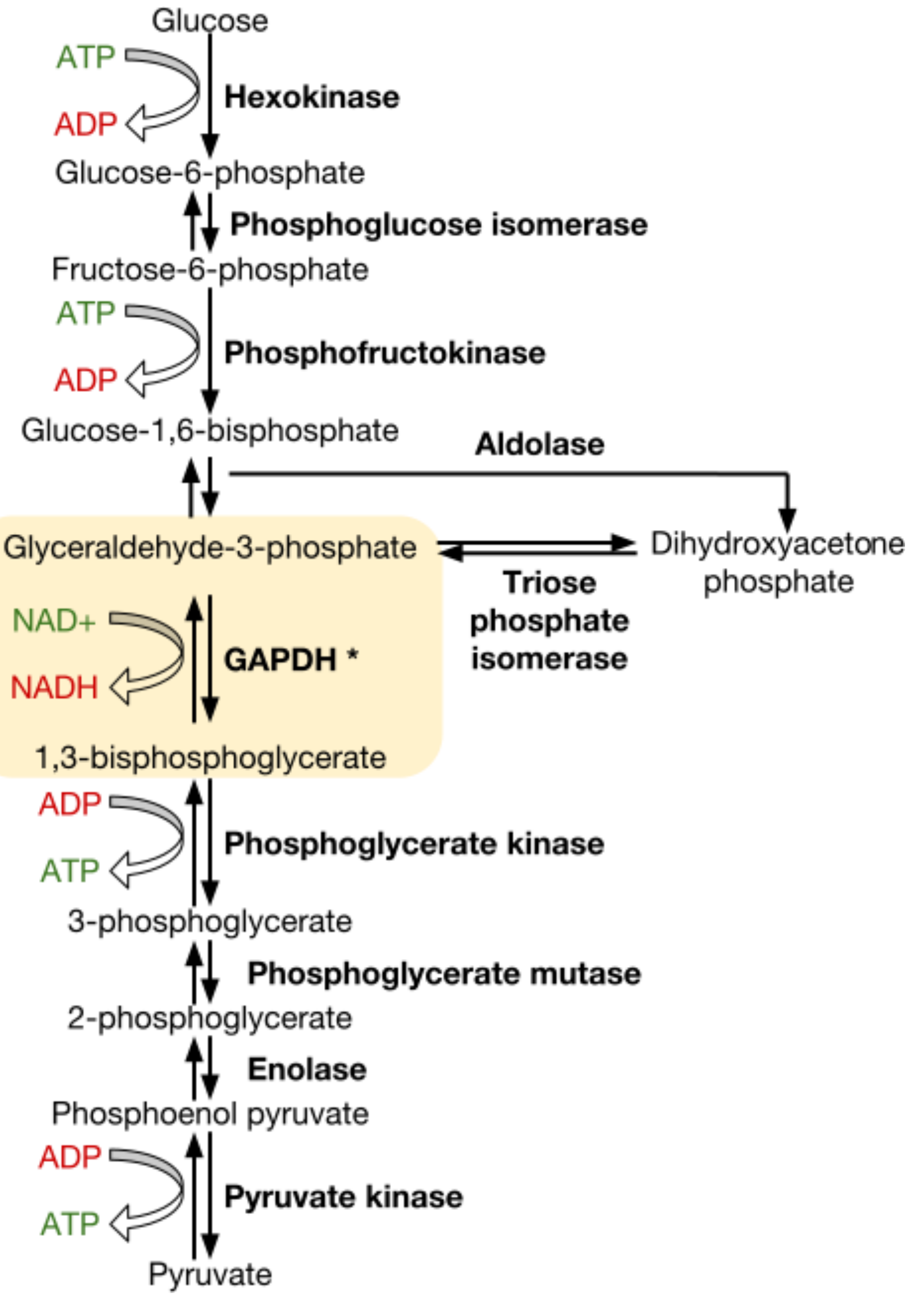

Supplementary Figure 1. Glycolysis. Simplified scheme for the core glycolytic pathway. Note that glyceraldehyde-3-phosphate dehydrogenase (GAPDH, marked with an asterisk), the sixth reaction after aldolase, is the first glycolytic step where energy (reducing power) is generated. Before that step, energy in the form of 2 ATP molecules is consumed; after that, energy is produced as 2 net ATP molecules per starting glucose equivalent. Note also that triose phosphate isomerase is responsible for converting dihydroxyacetone phosphate to a second glyceraldehyde-3-phosphate, which is further converted to 1,3-bisphosphoglycerate by GAPDH.

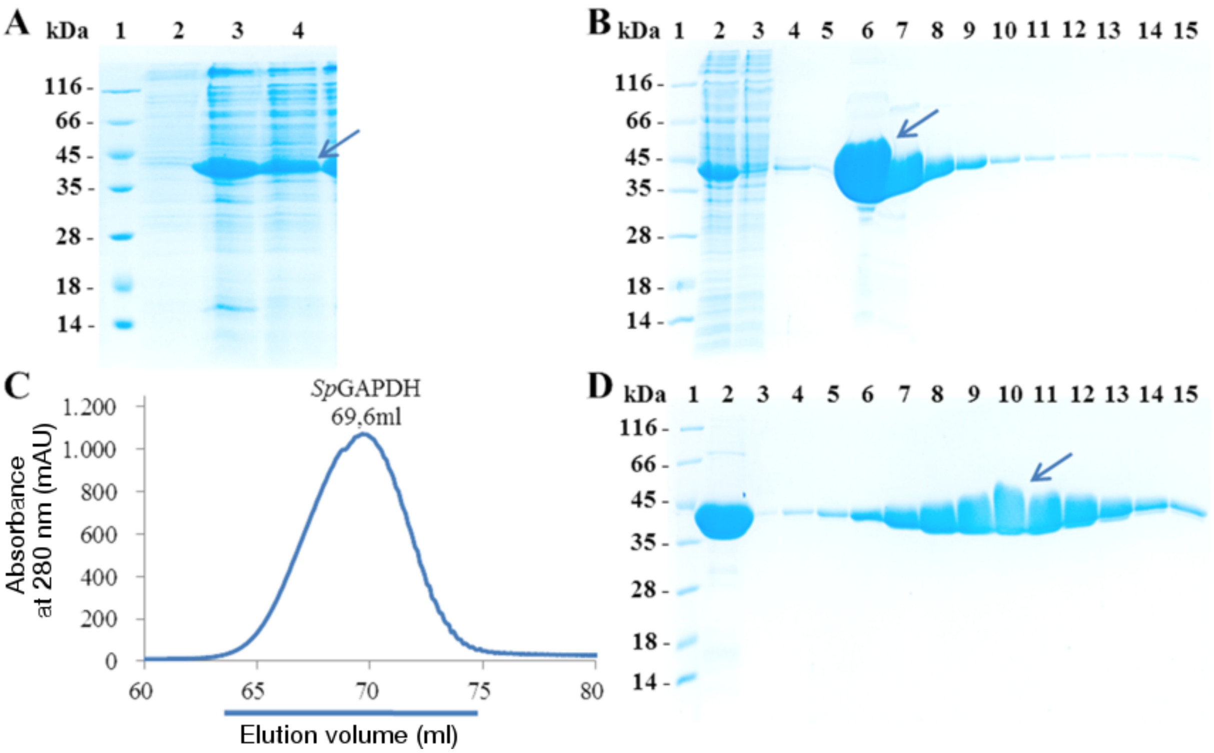

Supplementary Figure 2. Purification of *Sp*GAPDH. **A**, Coomassie Brilliant Blue (CBB)-stained SDS-PAGE of *Sp*GAPDH expression as a soluble protein. 1, molecular weight markers; 2, crude extract, uninduced control; 3, crude extract, induced with 0.5 mM IPTG at 20 ºC; 4, soluble fraction. An arrow points to the band containing *Sp*GAPDH. **B**, CBB-stained SDS-PAGE of *Sp*GAPDH purified by nickel affinity chromatography. 1, molecular weight markers; 2, input; 3, flow-through; 4, wash; 5-15, fractions eluted with buffer with 250 mM imidazole. **C**, Gel filtration chromatography of pooled fractions 5-12. *Sp*GAPDH elutes as a single peak whose molecular size corresponds to a homotetramer. The blue bar underneath the elution peak contains all fractions analyzed by SDS-PAGE. **D**, CBB-stained SDS-PAGE of fractions purified in **C**. 1, molecular weight markers; 2, input (fractions 5-12 from **B**); 3-15, fractions under the elution peak in **C**.

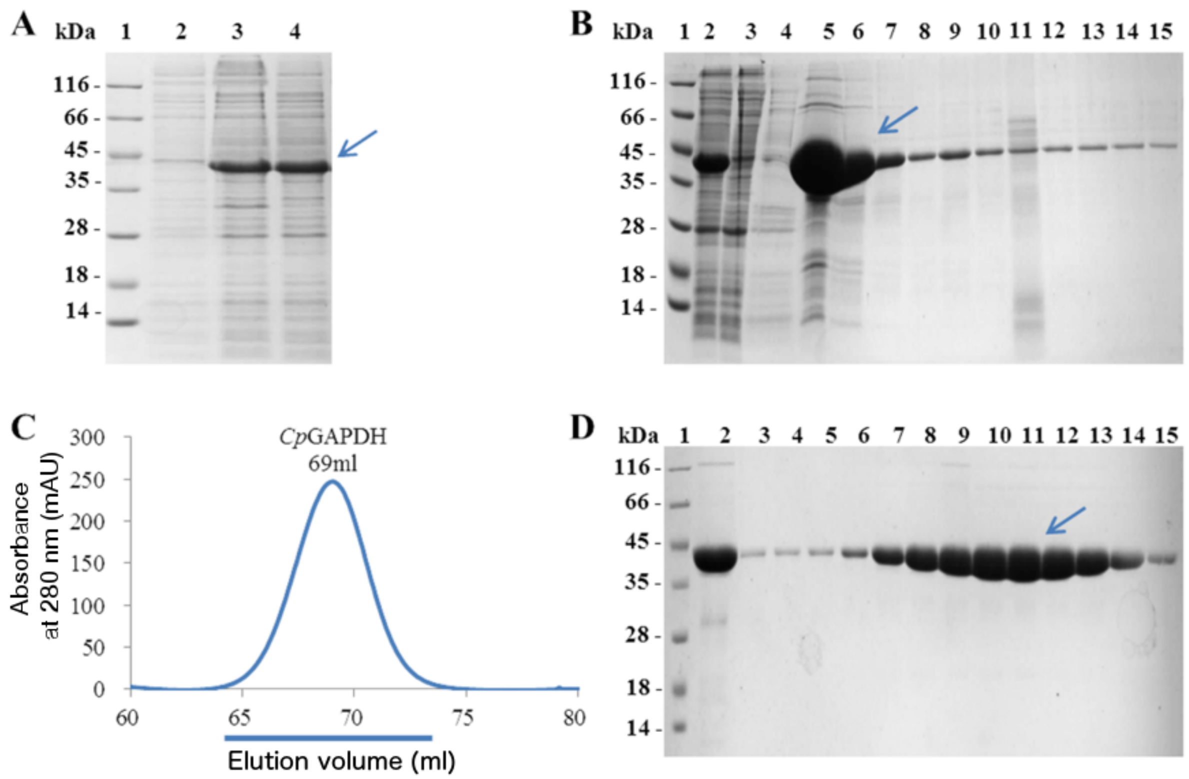

Supplementary Figure 3. Purification of *Cp*GAPDH. A, Coomassie Brilliant Blue (CBB)-stained SDS-PAGE of *Cp*GAPDH expression as a soluble protein. 1, molecular weight markers; 2, crude extract, uninduced control; 3, crude extract, induced with 0.5 mM IPTG at 20 ºC; 4, soluble fraction. An arrow points to the band containing *Cp*GAPDH. **B**, CBB-stained SDS-PAGE of *Cp*GAPDH purified by nickel affinity chromatography. 1, molecular weight markers; 2, input; 3, flow-through; 4, wash; 5-15, fractions eluted with buffer with 250 mM imidazole. **C**, Gel filtration chromatography of pooled fractions 5-15. *Cp*GAPDH elutes as a single peak whose molecular size corresponds to a homotetramer. The blue bar underneath the elution peak contains all fractions analyzed by SDS-PAGE. **D**, CBB-stained SDS-PAGE of fractions purified in **C**. 1, molecular weight markers; 2, input (fractions 5-15 from **B**); 3-15, fractions under the elution peak in **C**.

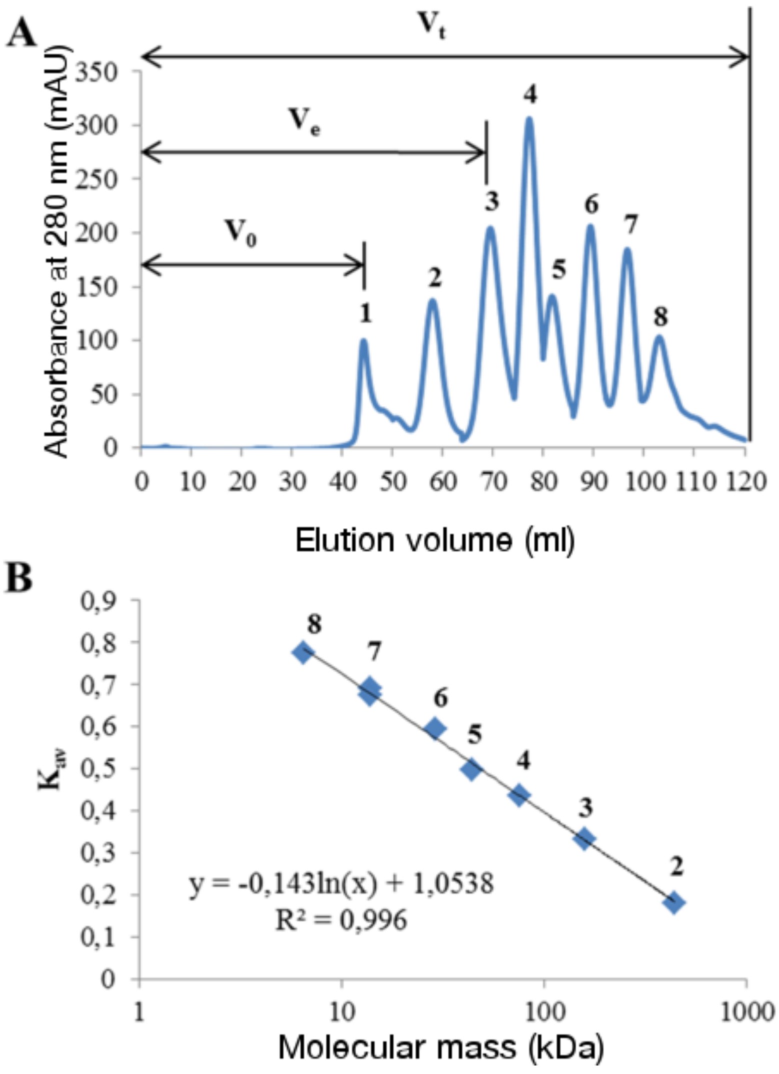

Supplementary Figure 4. Molecular size standard curve. A, A HiLoad 16/60 Superdex 200 column (GE Healthcare) was used for the final polishing of GAPDH from *S. pyogenes* (*Sp*GAPDH) and *C. perfringens* (*Cp*GAPDH). The column was calibrated using high and low molecular weight calibration kits by following the manufacturer's (GE Healthcare) instructions. **B**, **1**, Dextran blue (2000 kDa); **2**, ferritin (400 kDa); **3**, aldolase (158 kDa); **4**, conalbumin (75 kDa); **5**, ovalbumin (44 kDa); **6**, carbonic anhydrase (29 kDa); **7**, ribonuclease A (13.7 kDa); **8**, aprotinin (6.5 kDa). The partition coefficient $K_{av} = (V_e - V_0) / (V_t - V_0)$ was calculated assuming void volume V_0 = 40 ml and total volume V_t = 124 ml. V_e stands for elution volume. The data were fitted to the following regression formula: y = *a* ln(x) + *b*.

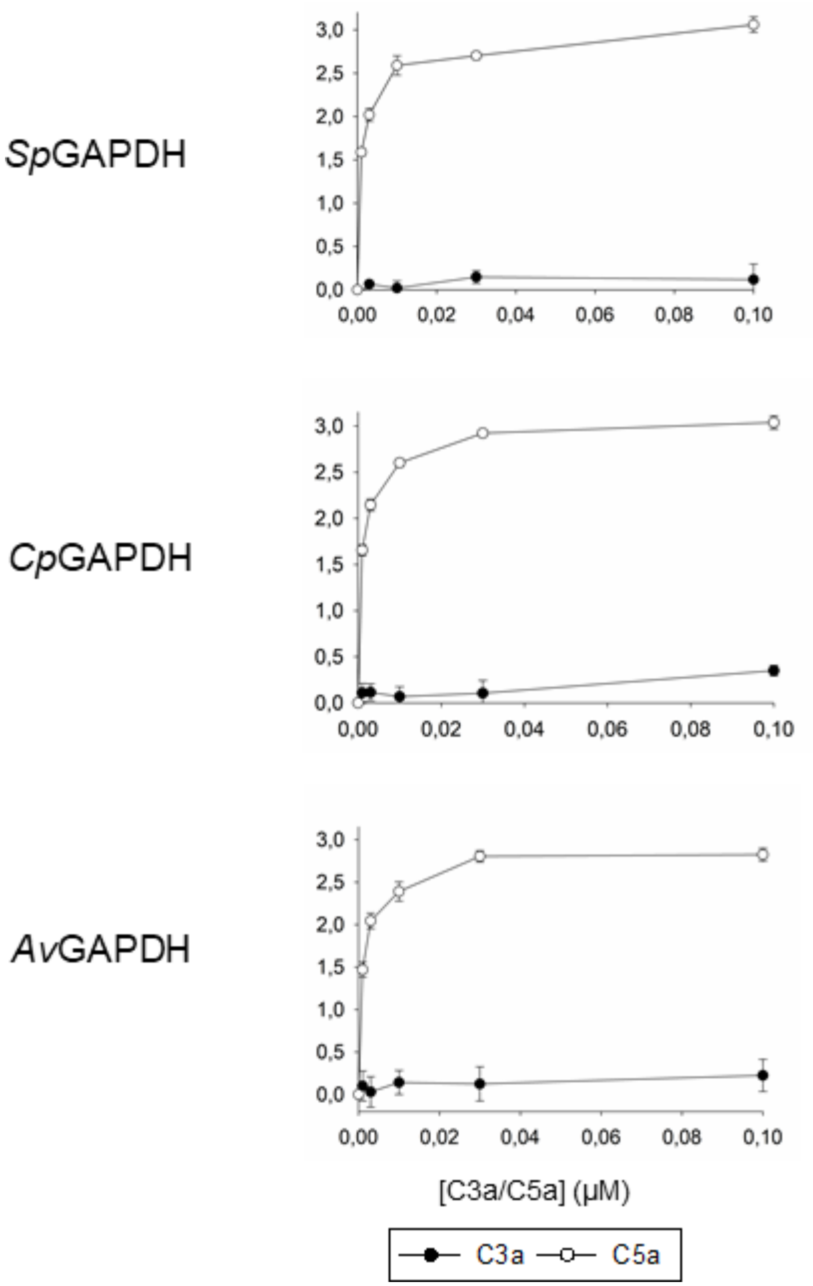

Supplementary Figure 5. ELISA interaction assay for C3a. Interaction of human complement factors C3a (black circles) in comparison with C5a (open circles) with surface-immobilized *Av*GAPDH, *Sp*GAPDH, and *Cp*GAPDH measured by an ELISA assay. Absorbance at 492 nm is plotted against increasing C3a or C5a concentration. Data points and error bars represent mean ± sd (standard deviation; $N = 3$). The plots show that C3a does not appreciably interact with any of the tested GAPDH enzymes from gram-positive bacterial pathogens, in contrast to C5a, for which a significant interaction is apparent.
